# Nutritional status, dietary antioxidant capacity, and dietary diversity in patients with newly diagnosed hematological malignancies: a case–control study

**DOI:** 10.1007/s00520-026-10817-5

**Published:** 2026-06-02

**Authors:** Busra Nur Koyuncu, Merve Yilmaz, Fatos Dilan Koseoglu

**Affiliations:** 1 Department of Nutrition and Dietetics (MSc), Graduate School of Health Sciences, Izmir Tinaztepe University, Izmir, Turkey; 2 Department of Nutrition and Dietetics, Faculty of Health Sciences, Izmir Tinaztepe University, Izmir, Turkey; 3 Department of Internal Medicine, Division of Hematology, Faculty of Medicine, Izmir Bakircay University, Izmir, Turkey

**Keywords:** Hematological malignancies, Dietary total antioxidant capacity, Nutritional status, Dietary diversity, Antioxidants

## Abstract

**Purpose:**

Nutritional status and dietary antioxidant capacity are important determinants of clinical resilience in cancer; however, data in hematological malignancies remain limited. We aimed to compare nutritional status, dietary total antioxidant capacity (DTAC), and dietary diversity between patients with newly diagnosed hematological malignancies and healthy controls.

**Methods:**

This case–control study included 34 patients with newly diagnosed hematological malignancies (12 lymphoma, 11 leukaemia, 11 multiple myeloma) and 34 age- and sex-matched healthy controls aged 18–65 years. DTAC was estimated using the ferric reducing antioxidant power (FRAP) method based on a validated food frequency questionnaire. Nutrient intakes and the dietary diversity score (DDS) were derived from 3-day dietary records. Nutritional status was assessed using the Global Leadership Initiative on Malnutrition (GLIM) criteria, along with fat-free mass index (FFMI), and mid-upper arm circumference (MUAC).

**Results:**

Patients had significantly lower DTAC levels than controls (3.85 ± 1.58 vs. 8.68 ± 3.21 mmol/day, *p* < 0.05). According to GLIM criteria, 35.3% of patients had moderate malnutrition and 26.5% had severe malnutrition, A declining trend in DTAC was observed with worsening malnutrition (*p* = 0.034), although this did not reach the significance threshold after multiplicity adjustment. Low muscle mass was more prevalent in patients as assessed by both FFMI and MUAC. Energy, protein, zinc, vitamin E, and folate intakes were significantly lower in patients (all *p* < 0.05), whereas DDS did not differ (*p* > 0.05). DTAC was positively correlated with intakes of coffee, whole-grain bread, green vegetables, citrus fruits, and fresh fruit (*p* ≤ 0.05).

**Conclusions:**

Significant differences in nutritional status and DTAC between patients and healthy individuals suggest that suboptimal dietary patterns may accompany hematological malignancies at diagnosis. These findings highlight the potential importance of adequate macro- and micronutrient intake; however, further prospective studies are needed to determine whether targeted nutritional interventions can improve clinical outcomes or contribute to long-term risk reduction.

## Introduction

Hematological malignancies, including leukaemia, lymphoma, and multiple myeloma arise from the bone marrow or lymphoid tissues and account for a substantial proportion of the global cancer burden. Their incidence is expected to increase as populations age and exposure to lifestyle and environmental risk factors accumulates [1, 2]. Nutritional status is an important determinant of treatment tolerance, complications, and survival in patients with cancer. In hematological malignancies, malnutrition is common due to tumour-related metabolic changes, systemic inflammation, and treatment-related side effects, and it adversely affects quality of life [3]. Reported prevalence rates of malnutrition in this population range from 17 to 43% [4]. The Global Leadership Initiative on Malnutrition (GLIM) provides a consensus-based framework for the diagnosis of malnutrition in adults, and scoping reviews indicate a wide but consistently high prevalence across oncology populations [5]. GLIM-based assessments in hematological malignancies have also reported variable prevalence rates; for example, 38.8% in patients with non-Hodgkin lymphoma [6].

Oxidative stress, defined as an imbalance between reactive oxygen species (ROS) and antioxidant defenses, contributes to carcinogenesis, although physiological ROS signaling remains essential for cellular regulation [7, 8]. Dietary antioxidants such as vitamins C and E, carotenoids, selenium, and polyphenols, derived from fruits, vegetables, whole grains, and coffee, can neutralize free radicals and modulate inflammation [9, 10]. Dietary total antioxidant capacity (DTAC) reflects the cumulative effect of these diet-derived antioxidants [11].

Meta-analyses have demonstrated inverse associations between higher DTAC and several cancers, including colorectal, pancreatic, endometrial, and breast cancer [12, 13]. Similarly, dietary diversity scores (DDS) and related food biodiversity metrics have been associated with long-term health and lower gastrointestinal cancer risk [14]. However, evidence on DTAC and dietary diversity in hematological malignancies remains limited, as most studies have focused on circulating biomarkers rather than dietary intake [15].

To date, no studies have simultaneously examined DTAC, dietary diversity, and nutritional status in newly diagnosed, treatment-naive patients with hematological malignancies. Although hematological malignancies such as leukaemia, lymphoma, and multiple myeloma are biologically heterogeneous, they share common inflammatory, metabolic, and treatment-related pathways that justify combined evaluation in clinical research, particularly at diagnosis [16]. Including a healthy control group enables assessment of whether nutritional impairments are already present at diagnosis, independent of treatment effects. Identifying such differences may help inform preventive strategies and optimize supportive care.

## Methods

### Study design and participants

This case–control study was conducted at the hematology outpatient clinic of a tertiary university hospital between January and August 2025. The patient group included 34 individuals aged 18–65 years with newly diagnosed leukaemia, lymphoma, or multiple myeloma, who had not yet initiated treatment. The control group comprised 34 age- and sex-matched healthy volunteers without a history of malignancy or chronic disease, recruited from the same region. Matching was performed at the group level based on age and sex. Participants were eligible if they could undergo bioelectrical impedance analysis (BIA), were literate, and provided informed consent. Exclusion criteria were prior cancer diagnosis or treatment, conditions precluding BIA (e.g., pacemaker use, severe edema, pregnancy), and unwillingness to participate. Sample size was calculated using G*Power (version 3.1.9.2) based on the independent samples t-test (two-tailed). Assuming a medium effect size (d = 0.50), α = 0.05, and power (1 − β) = 0.80, the required sample size was 34 participants per group (total n = 68) [17]. Written informed consent was obtained from all participants. The study was approved by the University Non-Interventional Research Ethics Committee (Approval No: IZTUMUKCE2025/39) and conducted in accordance with the Declaration of Helsinki.

### Data collection

Dietary total antioxidant capacity (DTAC) was estimated using the ferric reducing antioxidant power (FRAP) method based on a validated food frequency questionnaire (FFQ). Macro- and micronutrient intakes and dietary diversity score (DDS) were derived from 3-day dietary records. Nutritional status was evaluated using the GLIM criteria, along with fat-free mass index (FFMI), and mid-upper arm circumference (MUAC).

### Dietary total antioxidant capacity (DTAC) calculation

Long-term dietary intake was assessed using a semi-quantitative FFQ covering the previous six months. Reported consumption frequencies were converted into daily intakes (g/day) using standard portion sizes. Antioxidant values were assigned using a standardized database developed by Carlsen et al. [18] based on the FRAP method; no de novo laboratory analyses were performed. This database includes antioxidant capacity values for more than 3,100 foods. For each food item, the corresponding FRAP value (mmol/100 g) was multiplied by the estimated daily intake, and total DTAC was expressed as mmol/day [18].

### Dietary diversity score (DDS)

Dietary diversity was assessed using the dietary diversity score (DDS), an indicator of overall diet quality reflecting the variety of foods consumed across major food groups [19]. The DDS method was based on approaches described by the FAO and Kant et al. (1991), adapted to Turkish portion standards (TBSA, 2019). Five major food groups, grains, vegetables, fruits, meat and meat products, and dairy products, were considered, comprising 23 subgroups. Dietary intake was assessed using 3-day prospective dietary records (two weekdays and one weekend day), and DDS was calculated as the mean of these records. Records were collected during face-to-face interviews to ensure completeness and accuracy. A subgroup was considered “consumed” if at least half of the defined portion size was reported. Each major food group contributed up to 2 points, yielding a total DDS ranging from 0 to 10. Scores were categorized as low (< 3.5), moderate (3.5–6.5), or high dietary diversity (> 6.5) [20].

Dietary records were analyzed using the Nutrition Information System (BEBİS 9.0, Pasifik Company, 2016) to estimate mean daily energy and macro- and micronutrient intakes. Nutrient adequacy was evaluated according to the Recommended Dietary Allowances (RDA), and classified as inadequate (< 67% of RDA), adequate (67–133%), or excessive (> 133%) [21].

### Assessment of nutritional status

Nutritional status was evaluated in two steps. First, malnutrition risk was screened using the Malnutrition Universal Screening Tool (MUST), which assesses body mass index (BMI), unintentional weight loss over the past 3–6 months, and reduced food intake due to disease, yielding a score categorized as low (0), medium (1), or high risk (≥ 2) [22]. Individuals identified at risk were further assessed according to the GLIM criteria. A diagnosis of malnutrition required at least one phenotypic criterion (weight loss, low BMI, or reduced muscle mass assessed by FFMI) and one etiologic criterion (reduced intake/absorption or inflammation). In the patient group, the etiological criterion of inflammation was considered fulfilled due to presence of a newly diagnosed hematological malignancy, representing disease-related inflammation as defined by the GLIM framework. This approach is supported by a recent systematic review indicating that a cancer diagnosis may serve as a valid surrogate for inflammation within the GLIM framework. In the control group, the absence of clinical inflammation was confirmed by medical history; therefore, serum inflammatory biomarkers (e.g., C-reactive protein) were not collected to avoid unnecessary invasive procedures [5]. Body composition was measured using bioelectrical impedance analysis (BIA, TANITA BC-418 MA) under standardized conditions. Fat-free mass (FFM) was calculated and normalized for height to obtain FFMI (kg/m^2^), with cut-offs of < 17 kg/m^2^ for men and < 15 kg/m^2^ for women indicating reduced muscle mass, as recommended by the GLIM [5]. BMI was calculated as weight/height^2^ and classified according to World Health Organization criteria (2011). Waist circumference risk thresholds were ≥ 94 cm for men and ≥ 80 cm for women [23]. Mid-upper arm circumference (MUAC) was meaured at the midpoint between the acromion and olecranon; values < 24 cm in men and < 23 cm in women were considered indicative of malnutrition risk [24].

### Statistical analysis

Data were analysed using IBM SPSS Statistics 25.0 (IBM Corp., Armonk, NY, USA). Descriptive statistics were presented as mean ± standard deviation, median (min–max), or percentages. Group comparisons were performed using the Mann–Whitney U test or Kruskal–Wallis test for non-normally distributed variables, and the chi-square or Fisher’s exact test for categorical variables. Correlations were assessed using Spearman’s rank correlation coefficient. To account for potential confounding, analysis of covariance (ANCOVA) was performed to adjust for smoking status in DTAC comparisons. Homogeneity of variances was assessed using Levene’s test; when this assumption was violated (*p* < 0.05), Welch ANOVA with Games–Howell post hoc tests was applied. To control for Type I error in multiple comparisons, Bonferroni correction was applied for DTAC comparisons across GLIM categories (adjusted α = 0.016). For other nutritional and dietary parameters, results were interpreted within an exploratory framework. Statistical significance was set at *p* < 0.05.

## Results

### Participant characteristics

The mean age was 53.6 ± 9.3 years in the case group and 51.5 ± 8.9 years in the control group, with no significant difference between groups. The sex distribution was also similar. Smoking status differed significantly, with a higher proportion of current and former smokers among patients compared with controls. The mean duration of smoking was longer in patients than in controls (17.2 ± 15.0 vs. 6.2 ± 12.2 years, *p* = 0.001). Among patients, 35.3% were diagnosed with lymphoma, 32.4% with leukaemia, and 32.4% with multiple myeloma. Participant characteristics are presented in Table 1.

**Table 1:** Participant characteristics

Characteristics	Case Group (*n* = 34)		Control Group (*n *= 34)		Test*
	n	%	n	%	(χ^2^, *p*)
Sex					
Female	18	52.9	21	61.8	*χ^2^, *p* = 0.696
Male	16	47.1	13	38.2	*χ^2^, *p* = 0.469
Alcohol use					
Yes	12	35.3	8	23.5	*χ^2^, *p* = 0.763
No	14	41.2	17	50.0	*χ^2^, *p* = 0.747
Occasionally	8	23.5	9	26.5	
Smoking status					
Never smoked	11ᵃ	32.4	24ᵇ	70.6	*χ^2^ = 11.956
Quit	10	29.4	2	5.9	
Currently smoking	13	38.2	8	23.5	*p* = 0.003
Age (years)	53.55 ± 9.25	55.50 (25–65)	51.54 ± 8.91	51 (25–65)	**z = −1.033 *p* = 0.301
Duration of smoking (years)	17.20 ± 14.98	17.50 (0–45)	6.22 ± 12.19	0 (0–40)	**z = −3.325 *p* = 0.001

*Chi-square test, **Mann–Whitney U test. Different superscripts (a, b) indicate the source of group differences

### Nutritional status

As shown in Table 2, BMI and FFMI did not differ significantly between groups; however MUAC was lower in patients. The prevalence of low muscle mass according to FFMI classification was significantly higher in patients than in controls (32.4% vs. 11.8%, *p* = 0.023). Based on MUST, 67.6% of patients were at risk of malnutrition, whereas no risk was detected in controls (*p* < 0.001). Similarly, no malnutrition was detected in the control according to GLIM criteria.

**Table 2:** Nutritional status of participants

Nutritional characteristics	Case Group (*n* = 34) Median (min–max)/*n* (%)	Control Group (*n* = 34) Median (min–max)/*n* (%)	Test*
BMI (kg/m^2^)	26.3 (16.3–44.3)	26.8 (21.7–36.5)	Z = −0.600; *p* = 0.548
Waist circumference (cm)	94 (63–125)	90 (71–128)	Z = −0.288; *p* = 0.773
MUAC (cm)	29 (16–39)	31 (23–41)	Z = −2.414; *p* = 0.016
FFM (kg)	48.3 (14.8–71.2)	48.8 (33.9–75.3)	Z = −0.744; *p* = 0.457
FFMI (kg/m^2^)	17.95 (10.1–28.6)	18.1 (14.1–25.2)	Z = −0.552; *p* = 0.581
BMI classification			
• Underweight	1 (2.9%)	-	χ^2^ = 1.963; *p* = 0.637
• Normal	12 (35.3%)	15 (44.1%)	
• Overweight	16 (47.1%)	12 (35.3%)	
• Obese	5 (14.7%)	7 (20.6%)	
FFMI classification			
• Low muscle mass	11 (32.4%)	4 (11.8%)	χ^2^ = 7.523; *p* = 0.023
• Average muscle mass	19 (55.9%)	29 (85.3%)	
• Above average	4 (11.8%)	1 (2.9%)	
Malnutrition (MUST)			
• No risk	11 (32.4%)	34 (100%)	
• At risk	23 (67.6%)	-	χ^2^ = 35.515; *p* < 0.001
Malnutrition (GLIM)			
• No malnutrition	13 (38.2%)	-	
• Moderate	12 (35.3%)	-	
• Severe	9 (26.5%)	-	
• Total malnutrition	21 (61.8%)	-	
MUAC classification			
• No risk	26 (76.5%)	33 (97.1%)	
• At risk	8 (23.5%)	1 (2.9%)	χ^2^ = 4.417; *p* = 0.0

*Mann–Whitney U Test, Chi-square Test. *BMI* Body mass index, *MUAC* Mid-upper arm circumference, *FFM* Fat-free mass, *FFMI* Fat-free mass index, *MUST* Malnutrition Universal Screening Tool, *GLIM* Global Leadership Initiative on Malnutrition. Data are presented as median (min–max) or *n* (%)

### Dietary total antioxidant capacity (DTAC) and dietary diversity

As shown in Table 3, no significant differences were observed between patients and controls in DDS or in the distribution of low, moderate, and high dietary diversity categories. In contrast, DTAC levels were significantly lower in patients than in controls (3.85 ± 1.58 vs. 8.68 ± 3.21 mmol/day, *p* ≤ 0.001).

**Table 3:** Dietary total antioxidant capacity (DTAC) and dietary diversity (DDS) of participants

Variable	Cases (*n* = 34)	Controls (*n* = 34)	*p*-value
DTAC (mmol/day), mean ± SD	3.85 ± 1.58	8.68 ± 3.21	< 0.001
DDS (score), mean ± SD	4.02 ± 1.59	4.67 ± 1.62	0.123
DDS categories, *n* (%)			0.335
• Low diversity (< 3.5)	16 (47.1)	12 (35.3)	
• Moderate diversity (3.5–6.5)	15 (44.1)	15 (44.1)	
• High diversity (> 6.5)	3 (8.8)	7 (20.6)	

Values are presented as mean ± standard deviation (SD) or *n* (%). *p*-values were obtained using Mann–Whitney U test and chi-square test. *DTAC* dietary total antioxidant capacity, *DDS* dietary diversity score

As shown in Table 4, DTAC levels were significantly higher in controls than in patients, including those without malnutrition risk according to MUST (*p* < 0.001). Among patients, DTAC differed across GLIM categories (Kruskal–Wallis *p* = 0.034); however, pairwise comparisons with Bonferroni correction (α = 0.016) were not significant. Due to unequal variances (Levene’s test *p* = 0.004), Welch ANOVA was applied and confirmed higher DTAC levels in controls (*p* < 0.001). This difference remained significant after adjustment for smoking status using ANCOVA (*p* < 0.001).

**Table 4:** DTAC levels according to nutritional status

Nutritional status	Case group (*n* = 34) Mean ± SD/Median (min–max)	Control group (*n* = 34) Mean ± SD/Median (min–max)	Test*
MUST			
• No risk	4.05 ± 0.92/4.13 (2.38–5.53)	8.68 ± 3.21/8.75 (2.59–14.80)	Z = −3.901; *p* < 0.001^1^
GLIM			
• No malnutrition	7.38 ± 0.52/7.38 (7.01–7.74)	-	-
• Moderate malnutrition	4.09 ± 1.57/3.91 (1.76–6.58)	-	-
• Severe malnutrition	3.18 ± 1.81/2.44 (1.24–5.99)	-	-
Test			*H* = 6.776; *p* = 0.034^2^

*MUST* Malnutrition Universal Screening Tool, *GLIM* Global Leadership Initiative on Malnutrition, *DTAC* Dietary total antioxidant capacity. *^1^Mann–Whitney U Test; ^2^Kruskal–Wallis H Test. Levene’s test indicated unequal variances (F = 9.049, *p* = 0.004); comparisons were performed using Welch ANOVA with Games-Howell post-hoc tests. Results remained significant (*p* < 0.001) after adjusting for smoking status (ANCOVA).* Statistical significance was set at *p* < 0.016 following Bonferroni correction (0.05/3 groups) to control for Type I error

According to RDA-based evaluation (Table 5), energy, carbohydrate (%), protein (g), zinc, vitamin E, and folate intakes were significantly lower in patients, whereas other nutrients did not differ between groups (*p* > 0.05). Correlation analyses (Table 6) showed that, in patients, DTAC was positively correlated with energy intake, protein intake (g/day and % energy), and vitamin E (r = 0.296–0.464), and negatively correlated with calcium intake. DTAC was also positively associated with the consumption of legumes, olive oil, nuts, vegetables (particularly green vegetables), fruits (including citrus), whole-grain bread, herbal tea, and coffee, with the strongest correlation observed for coffee (r = 0.719).

**Table 5:** Comparison of daily energy and nutrient intakes according to RDA requirements

	Case group (*n* = 34)						Control group (*n* = 34)						*Test*
	Inadequate		Adequate		Excessive		Inadequate		Adequate		Excessive		
	*n*	*%*	*n*	*%*	*n*	*%*	*n*	*%*	*n*	*%*	*n*	*%*	
Energy and nutrients	14	41.2	18	52.9	2	5.9	4	11.8	28	82.4	2	5.9	χ^2^ = 6.478; *p* = 0.028
Energy (kcal/day)	5	14.7	14	41.2	15	44.1	3	8.8	15	44.1	16	47.1	χ^2^ = 0.681; *p* = 0.793
Carbohydrate (g/day)	6	17.6	22	64.7	6	17.6	10	29.4	24	70.6	-	-	χ^2^ = 7.232; *p* = 0.022
Protein (g/day)	7	20.6	20	58.8	7	20.6	1	2.9	28	82.4	5	14.7	χ^2^ = 6.368; *p* = 0.038
Protein (%)	3	8.8	27	79.4	4	11.8	4	11.8	27	79.4	3	8.8	χ^2^ = 0.398; *p* = 1.000
Fat (%) kkal	4	11.8	11	32.4	19	55.9	-	-	12	35.3	22	64.7	χ^2^ = 4.094; *p* = 0.155
Linoleic acid (% energy)	1	2.9	5	14.7	28	82.4	5	14.7	8	23.5	21	61.8	χ^2^ = 4.577; *p* = 0.098
Alpha-linolenic acid (% energy)	5	14.7	12	35.3	17	50.0	4	11.8	12	35.3	18	52.9	χ^2^ = 0.277; *p* = 0.945
Fiber (g/day)	21	61.8	13	38.2	-	-	18	52.9	14	41.2	2	5.9	χ^2^ = 1.771; *p* = 0.554
Calcium (mg/day)	22	64.7	9	26.5	3	8.8	16	47.1	17	50	1	2.9	χ^2^ = 3.977; *p* = 0.159
Iron (mg/day)	14	41.2	19	55.9	1	2.9	7	20.6	22	64.7	5	14.7	χ^2^ = 4.302; *p* = 0.102
Magnesium (mg/day)	23	67.6	10	29.4	1	2.9	18	52.9	16	47.1	-	-	χ^2^ = 2.661; *p* = 0.216
Phosphorus (mg/day)	4	11.8	12	35.3	18	52.9	1	2.9	8	23.5	25	73.5	χ^2^ = 3.194; *p* = 0.181
Potassium (mg/day)	-	-	-	-	34	100.0	-	-	-	-	34	100	-
Sodium (mg/day)	15	44.1	16	47.1	3	8.8	12	35.3	16	47.1	6	17.6	χ^2^ = 1.117; *p* = 0.637
Zinc (mg/day)	13	38.2	11	32.4	10	29.4	12	35.3	20	58.8	2	5.9	χ^2^ = 7.899; *p* = 0.017
Copper (mg/day))	19	55.9	14	41.2	1	2.9	25	73.5	9	26.5	-	-	χ^2^ = 2.390; *p* = 0.257
Vitamin A (µg RE/day)	17	50.0	11	32.4	6	17.6	14	41.2	13	38.2	7	20.6	χ^2^ = 0.753; *p* = 0.773
Vitamin D (µg/day)	27	79.4	3	8.8	4	11.8	33	97.1	-	-	1	2.9	χ^2^ = 5.039; *p* = 0.068
Vitamin E (mg/day)	18	52.9	10	29.4	6	17.6	19	55.9	15	44.1	-	-	χ^2^ = 7.156; *p* = 0.024
Vitamin K (µg/day)	18	52.9	11	32.4	5	14.7	19	55.9	10	29.4	5	14.7	χ^2^ = 0.214; *p* = 0.944
Vitamin C (mg/day)	20	58.8	7	20.6	7	20.6	18	52.9	13	38.2	3	8.8	χ^2^ = 3.312; *p* = 0.202
Thiamin (mg/day)	2	5.9	6	17.6	26	76.5	-	-	8	23.5	26	76.5	χ^2^ = 2.215; *p* = 0.354
Riboflavin (mg/day)	17	50.0	15	44.1	2	5.9	15	44.1	18	52.9	1	2.9	χ^2^ = 0.715; *p* = 0.782
Vitamin B6 (mg/day)	24	70.6	10	29.4	-	-	18	52.9	14	41.2	2	5.9	χ^2^ = 2.851; *p* = 0.191
Vitamin B12 (µg/day)	18	52.9	8	23.5	8	23.5	19	55.9	10	29.4	5	14.7	χ^2^ = 1.018; *p* = 0.649
Folate (µg/day)	21	61.8	12	35.3	1	2.9	9	26.5	24	70.6	1	2.9	χ^2^ = 8.071; *p* = 0.011
Niacin (mg/day NE)	4	11.8	10	29.4	20	58.8	2	5.9	14	41.2	18	52.9	χ^2^ = 1.338; *p* = 0.545

Values are expressed as *n* (%). Group differences were assessed by the Chi-square (χ^2^) test; Fisher’s Exact test was used when appropriate. *p* < 0.05 was considered statistically significant. *RDA* Recommended Dietary Allowances

**Table 6:** Correlations of DTAC with nutrient intakes and food groups

Variable	Cases (*n* = 34) r/p	Controls (*n* = 34) r/p
Macronutrients and Micronutrients		
Energy (kcal/day)	0.331/0.028	−0.123/0.240
Carbohydrate (g/day)	0.241/0.085	0.034/0.424
Carbohydrate (% energy)	−0.183/0.150	−0.018/0.460
Protein (g/day)	0.464/0.003**	0.111/0.263
Protein (% energy)	0.296/0.044*	0.150/0.195
Fat (% energy)	0.134/0.225	−0.016/0.463
Saturated fatty acids (%)	−0.146/0.205	0.156/0.186
Linoleic acid (% energy)	−0.138/0.219	0.033/0.425
Alpha-linolenic acid (% energy)	−0.075/0.337	0.036/0.419
Fiber (g/day)	0.134/0.224	0.160/0.180
Calcium (mg/day)	−0.402/0.009**	−0.155/0.187
Iron (mg/day)	0.146/0.204	−0.120/0.249
Magnesium (mg/day)	0.026/0.442	0.026/0.441
Phosphorus (mg/day)	0.103/0.282	0.132/0.225
Potassium (mg/day)	0.159/0.184	−0.159/0.181
Sodium (mg/day)	0.021/0.453	−0.055/0.378
Zinc (mg/day)	−0.051/0.387	0.017/0.462
Copper (mg/day)	0.162/0.180	−0.314/0.033*
Vitamin A (µg RE/day)	0.162/0.180	0.068/0.350
Vitamin D (µg/day)	−0.052/0.384	0.120/0.246
Vitamin E (mg/day)	0.414/0.007**	−0.070/0.344
Vitamin K (µg/day)	0.087/0.312	0.151/0.193
Vitamin C (mg/day)	0.242/0.084	0.123/0.241
Thiamin (mg/day)	0.200/0.129	−0.043/0.402
Riboflavin (mg/day)	−0.001/0.497	−0.150/0.194
Vitamin B6 (mg/day)	−0.168/0.172	0.006/0.486
Vitamin B12 (µg/day)	−0.129/0.233	0.058/0.370
Folate (µg/day)	−0.001/0.498	−0.088/0.308
Niacin (mg NE/day)	0.125/0.241	0.214/0.108
Food Groups		
Milk (g/day)	0.013/0.471	−0.145/0.203
Yogurt (g/day)	0.131/0.231	0.076/0.333
Cheese (g/day)	0.187/0.144	0.315/0.033
Red meat (g/day)	−0.142/0.212	−0.152/0.192
Poultry (g/day)	0.100/0.287	−0.041/0.408
Fish and seafood (g/day)	−0.012/0.474	0.174/0.159
Organ meats (g/day)	0.239/0.086	−0.093/0.298
Processed meat (g/day)	0.182/0.152	0.104/0.276
Eggs (g/day)	0.279/0.055	0.373/0.014*
Legumes (g/day)	0.288/0.049*	0.074/0.337
Green vegetables (g/day)	0.458/0.003**	0.571/≤ 0.001**
Vegetables (g/day)	0.520/0.001**	0.632/≤ 0.001**
Citrus fruits (g/day)	0.432/0.005*	0.206/0.118
Fresh fruits (g/day)	0.347/0.022	0.535/≤ 0.001**
Fruit juice (ml/day)	0.331/0.028	0.210/0.113
White bread (g/day)	−0.165/0.176	−0.132/0.225
Whole grain bread (g/day)	0.580/≤ 0.001**	0.391/0.010*
Rice, bulgur, pasta (g/day)	0.197/0.131	−0.018/0.458
Cakes, pastries, biscuits (g/day)	−0.246/0.080	0.081/0.323
Olive oil (g/day)	0.296/0.045*	0.196/0.129
Sunflower oil (g/day)	0.069/0.349	−0.217/0.105
Margarine (g/day)	0.193/0.137	−0.135/0.219
Olives (g/day)	0.253/0.075	0.247/0.076
Butter (g/day)	0.088/0.310	0.311/0.035*
Packaged fruit juice (g/day)	−0.081/0.325	−0.273/0.056
Nuts (g/day)	0.292/0.047*	0.408/0.007*
Honey, jam, molasses (g/day)	−0.236/0.089	−0.062/0.362
Coffee (ml/day)	0.719/≤ 0.0001**	0.258/0.067
Black tea (ml/day)	0.099/0.289	0.472/0.002*
Herbal tea (ml/day)	0.360/0.018*	0.135/0.220
Soft drinks (ml/day)	0.183/0.150	−0.170/0.165
Alcohol (ml/day)	−0.008/0.482	−0.039/0.412

*DTAC* Dietary total antioxidant capacity, *Spearman correlation analysis. *p < 0.05, **p < 0.01

In controls, DTAC was positively correlated with the intake of cheese, eggs, vegetables, fruits, whole-grain bread, nuts, butter, and black tea, with moderate correlation strength.

## Discussion

According to the GLIM criteria, malnutrition was highly prevalent at diagnosis among patients with hematological malignancies (35.3% moderate and 26.5% severe), whereas it was absent in the control group. This finding indicates that nutritional compromise is already present at diagnosis and highlights the vulnerability of this patient population. Early identification of malnutrition supports the need for routine nutritional screening and timely supportive interventions at diagnosis. While GLIM provides a standardized framework, comprehensive nutritional assessment in complex patient populations may benefit from integration with biochemical, hematological, and immunological parameters [25].

The prevalence observed in our study appears higher than in some previous reports. For example, Bian et al. reported a malnutrition prevalence of 44% across cancer patients, while Zou et al. found 38.8% in non-Hodgkin lymphoma [6, 26]. Similarly, GLIM-defined malnutrition was reported in 25.8% of hospitalized hematological patients [27]These differences may reflect variations in cancer type, disease stage and patient characteristics. Notably, our assessment was performed prior to treatment initiation, allowing evaluation of disease-related effects on nutritional status.

Reduced muscle mass was a prominent finding, with 32.4% of patients presenting with low FFMI compared to 11.8% in controls. This suggests an early decline in muscle reserves before treatment, consistent with previous studies linking muscle depletion to disease burden, systemic inflammation, and catabolic processes [28–30]. Importantly, reduced muscle mass has been associated with poorer overall and progression-free survival, underscoring its prognostic relevance and the importance of early nutritional and supportive interventions [31, 32]. Nutrient intake patterns further support the early nutritional deficits in patients. Compared with controls, patients had lower energy and protein intake, which may predispose them to malnutrition and reduced treatment tolerance. In addition, a greater proportion of total energy was derived from carbohydrates, suggesting an imbalanced macronutrient distribution and overall poorer diet quality.

Consistent with previous studies, insufficient protein intake is associated with muscle wasting and poorer survival outcomes [33, 34]. Micronutrient inadequacies were also evident, particularly for zinc, vitamin E, and folate, nutrients essential for immune function, redox balance, and cellular metabolism [35–37]. Lower intake of these micronutrients may further compromise antioxidant defense and immune competence. This is biologically plausible, as nutritional status influences oxidative stress pathways and adipokine regulation, potentially contributing to a pro-oxidative metabolic environment in malnourished individuals [38]. Beyond individual nutrients, patients exhibited significantly lower DTAC than controls. Although evidence in hematological malignancies is limited, higher DTAC has been associated with reduced risk of several cancers, including prostate, colorectal, and lung cancer [39–41]. The lower DTAC observed in patients may partly reflect reduced consumption of antioxidant-rich foods such as vegetables, fruits, and coffee. Given that dietary antioxidant capacity contributes to metabolic stability and treatment tolerance, the coexistence of low DTAC and malnutrition at diagnosis may represent a combined nutritional vulnerability. Consistent with this, poorer nutritional status was associated with lower DTAC, suggesting a link between malnutrition severity and reduced dietary antioxidant capacity.

Smoking was more prevalent among patients and may have contributed to increased oxidative stress. However, lower DTAC levels remained significant after adjustment for smoking, indicating that suboptimal dietary intake is an independent contributor to reduced antioxidant capacity [42, 43]. Although DTAC tended to decrease with worsening GLIM-defined malnutrition, this trend did not reach statistical significance, likely due to the limited sample size within subgroups.

Dietary diversity did not differ significantly between groups; however, nearly half of the patients exhibited low diversity, reflecting suboptimal dietary patterns. While a diverse diet is generally associated with higher intake of antioxidants (e.g., polyphenols and vitamins) that support redox balance, evidence regarding its relationship with cancer outcomes remains inconsistent. Some studies suggest protective effects of diverse consumption of fruits, vegetables, and dairy products [14, 44, 45], whereas others report no significant associations with cancer risk [46]. Importantly, DDS reflects dietary variety rather than nutrient adequacy. In our study, the the absence of differences in DDS masked underlying inadequacies in energy, protein, and micronutrient intake among patients, suggesting that DDS alone may be insufficient to evaluate nutritional status in hematological malignancies. Similarly, although controls consumed more antioxidant-rich foods, this was not fully captured by DDS, indicating that the score may not adequately reflect intake quantity or overall diet quality.

The positive association between DTAC and dietary diversity is consistent with findings in other populations, suggesting that greater dietary variety may enhance antioxidant intake [47]. These findings highlight the potential importance of improving both dietary quality and diversity to support antioxidant defense in this patient group.

### Strengths and limitations

The main strength of this study is the simultaneous evaluation of nutritional status, DTAC, and DDS in patients with hematological malignancies, a combination that has not been previously examined in this population at diagnosis.

However, several limitations should be considered. First, the case group included biologically and clinically distinct malignancies (leukaemia, lymphoma, and multiple myeloma). Although this approach reflects real-world hematology practice, the sample size did not allow formal subgroup analyses. Therefore, the findings should be interpreted as representing shared metabolic and inflammatory features at diagnosis rather than disease-specific effects. Second, the case–control design allows identification of associations but precludes causal inference. Third, although analyses were adjusted for smoking, residual confounding cannot be excluded, particularly given the higher prevalence of smoking among patients. Due to the limited sample size, adjustment was restricted to key confounders. Fourth, DTAC was estimated using FFQ-based dietary data and the FRAP method, which reflects dietary antioxidant exposure rather than in vivo antioxidant status. In addition, FFQ-based assessments may be subject to recall bias, and the FRAP method does not account for differences in bioavailability or all antioxidant mechanisms. DTAC values may also be influenced by high-consumption items such as coffee. Finally, the single-center design and reliance on self-reported dietary records may limit generalizability. Given the number of nutritional comparisons performed, the analyses should be considered exploratory and interpreted with caution regarding potential Type I error.

### Clinical implications and future directions

Our findings suggest that malnutrition and inadequate dietary antioxidant intake are already present at diagnosis in patients with hematological malignancies. These results support the integration of early nutritional screening and timely dietitian involvement into routine clinical care at diagnosis. Early nutritional assessment, along with strategies to improve overall diet quality and intake of antioxidant-rich foods, may contribute to better treatment tolerance and clinical outcomes. Personalized nutritional counseling and ongoing supportive monitoring may help address the early nutritional vulnerability observed in this population.

Future longitudinal and interventional studies with larger, disease-specific cohorts are needed to confirm these findings and to further explore the role of dietary antioxidant capacity in clinical outcomes, including survival and quality of life.

## Conclusıon

Newly diagnosed patients with hematological malignancies had lower DTAC, higher prevalence of malnutrition, and more frequent inadequacies in energy, protein, and key micronutrients compared with healthy controls. Although dietary diversity scores did not differ significantly, reduced diet quality and lower muscle mass indicated early nutritional vulnerability at diagnosis. These findings support the importance of early nutritional assessment and timely supportive interventions in this patient population. While the results suggest a potential role for dietary antioxidant capacity in maintaining redox balance, the observational design precludes causal inference. Future prospective and interventional studies are needed to confirm these associations and to evaluate the impact of early nutritional strategies on clinical outcomes.

## Data Availability

No datasets were generated or analysed during the current study.

## References

[1] Zhang N, Wu J, Wang Q, Liang Y, Li X, Chen G et al (2023) Global burden of hematologic malignancies and evolution patterns over the past 30 years. Blood Cancer J. 10.1038/s41408-023-00853-3 37193689 10.1038/s41408-023-00853-3 PMC10188596

[2] Lim EA, Ruffle JK, Gnanadurai R, Lee H, Escobedo-Cousin M, Wall E et al (2022) Differentiating central nervous system infection from disease infiltration in hematological malignancy. Sci Rep. 10.1038/s41598-022-19769-2 36138051 10.1038/s41598-022-19769-2 PMC9499957

[3] Ferlay J, Ervik M, Lam F, Laversanne M, Colombet M, Mery L, et al (2024) Global cancer observatory: cancer today. Lyon, France: International agency for research on cancer. Available from: https://gco.iarc.who.int/today

[4] Lu G, Li Q (2024) The controlling nutritional status score as a predictor of survival in hematological malignancies: a systematic review and meta-analysis. Front Nutr 11:1402328. 10.3389/fnut.2024.1402328 38938670 10.3389/fnut.2024.1402328 PMC11208478

[5] Cederholm T, Jensen GL, Correia MITD, Gonzalez MC, Fukushima R, Higashiguchi T et al (2019) GLIM criteria for the diagnosis of malnutrition - a consensus report from the global clinical nutrition community. Clin Nutr 38(1):1–9. 10.1016/j.clnu.2018.08.002 30181091 10.1016/j.clnu.2018.08.002

[6] Zou Y, Xu H, Lyu Q et al (2023) Malnutrition diagnosed by GLIM criteria better predicts long-term outcomes for patients with non-Hodgkin’s lymphoma: a prospective multicenter cohort study. Hematol Oncol 41(3):371–379. 10.1002/hon.3107 36416610 10.1002/hon.3107

[7] Domka K, Goral A, Firczuk M (2020) cROSsing the line: between beneficial and harmful effects of reactive oxygen species in B-cell malignancies. Front Immunol. 10.3389/fimmu.2020.01538 32793211 10.3389/fimmu.2020.01538 PMC7385186

[8] Preiser JC (2012) Oxidative stress. JPEN J Parenter Enteral Nutr 36(2):147–154. 10.1177/0148607111434963 22301329 10.1177/0148607111434963

[9] Lee SJ, Jeong JH, Lee IH, Lee J, Jung JH, Park HY et al (2019) Effect of high-dose vitamin C combined with anti-cancer treatment on breast cancer cells. Anticancer Res 39(2):751–758. 10.21873/anticanres.13172 30711954 10.21873/anticanres.13172

[10] Cancemi G, Cicero N, Allegra A, Gangemi S (2023) Effect of diet and oxidative stress in the pathogenesis of lymphoproliferative disorders. Antioxidants (Basel) 12(9):1674. 10.3390/antiox12091674 37759977 10.3390/antiox12091674 PMC10525385

[11] Ha K, Liao LM, Sinha R, Chun OK (2023) Dietary total antioxidant capacity, a diet quality ındex predicting mortality risk in US adults: evidence from the NIH-AARP diet and health study. Antioxidants. 10.3390/antiox12051086 37237952 10.3390/antiox12051086 PMC10215257

[12] Zamani B, Daneshzad E, Azadbakht L (2019) Dietary total antioxidant capacity and risk of gastrointestinal cancers: a systematic review and meta-analysis of observational studies. Arch Iran Med 22(6):328–335 31356099

[13] Abbasalizad Farhangi M, Vajdi M (2020) Dietary total antioxidant capacity (TAC) significantly reduces the risk of site-specific cancers: an updated systematic review and meta-analysis. Nutr Cancer. 10.1080/01635581.2020.1771385 32462920 10.1080/01635581.2020.1771385

[14] Huybrechts I, Chimera B, Hanley-Cook GT, Biessy C, Deschasaux-Tanguy M, Touvier M et al (2024) Food biodiversity and gastrointestinal cancer risk in nine European countries: analysis within a prospective cohort study. Eur J Cancer. 10.1016/j.ejca.2024.114258 39168001 10.1016/j.ejca.2024.114258

[15] Tsamesidis I, Pantaleo A, Pekou A, Gusani A, Iliadis S, Makedou K et al (2019) Correlation of oxidative stress biomarkers and hematological parameters in blood cancer patients from Sardinia, Italy. Int J Hematol Oncol Stem Cell Res 13(2):49–57 31372197 PMC6660479

[16] Kato S, Monna-Oiwa M, Andoh S, Oda Y, Nannya Y, Takahashi S et al (2025) The composite health risk assessment model (CHARM) predicts overall mortality and relapse, but not non-relapse mortality, in adults following unrelated single-unit cord blood transplantation. Turk J Hematol 42(3):181–195. 10.4274/tjh.galenos.2024.2025.0125 10.4274/tjh.galenos.2024.2025.0125 PMC12400884 40497704

[17] De Quadros Camargo C, Da Silva Borges D, De Oliveira PF, Chagas TR, Del Moral JAG, Durigon GS et al (2015) Individuals with hematological malignancies before undergoing chemotherapy present oxidative stress parameters and acute phase proteins correlated with nutritional status. Nutr Cancer 67(3):463–471. 10.1080/01635581.2015.1004732 25710080 10.1080/01635581.2015.1004732

[18] Carlsen MH, Halvorsen BL, Holte K, Bøhn SK, Dragland S, Sampson L et al (2010) The total antioxidant content of more than 3100 foods, beverages, spices, herbs and supplements used worldwide. Nutr J 9:3. 10.1186/1475-2891-9-3 20096093 10.1186/1475-2891-9-3 PMC2841576

[19] Zhang J, Zhao A (2021) Dietary diversity and healthy aging: a prospective study. Nutrients. 10.3390/nu13061787 34073820 10.3390/nu13061787 PMC8225052

[20] Kant AK, Schatzkin A, Harris TB, Ziegler RG, Block G (1993) Dietary diversity and subsequent mortality in the first National Health and Nutrition Examination Survey epidemiologic follow-up study. Am J Clin Nutr 57(3):434–440. 10.1093/ajcn/57.3.434 8382446 10.1093/ajcn/57.3.434

[21] Institute of Medicine (US) (2006) Dietary reference ıntakes. National Academies Press, Washington (DC). 10.17226/11537

[22] Boléo-Tomé C, Monteiro-Grillo I, Camilo M, Ravasco P (2012) Validation of the malnutrition universal screening tool (MUST) in cancer. Br J Nutr 108(2):343–348. 10.1017/S000711451100571X 22142968 10.1017/S000711451100571X

[23] World Health Organization (2008) Waist circumference and waist-hip ratio: report of a WHO expert consultation. Geneva 8–11:2011

[24] Jung EH, Hiratsuka Y, Suh SY, Won SH, Choi SE, Kang B et al (2022) Association between mid-upper arm circumference and functional status in patients with advanced cancer. Clin Nutr Open Sci 45:72–79. 10.1016/j.nutos.2022.08.002

[25] Mądra-Gackowska K, Szewczyk-Golec K, Gackowski M, Hołyńska-Iwan I, Parzych D, Czuczejko J, et al. (2025) Selected biochemical, hematological, and ımmunological blood parameters for the ıdentification of malnutrition in polish senile ınpatients: a cross-sectional study. J Clin Med. 14(5). 10.3390/jcm14051494. 10.3390/jcm14051494 PMC11900367 40094974

[26] Bian W, Li Y, Wang Y, Chang L, Deng L, Li Y et al (2023) Prevalence of malnutrition based on Global Leadership Initiative in Malnutrition criteria for completeness of diagnosis and future risk of malnutrition based on current malnutrition diagnosis: systematic review and meta-analysis. Front Nutr. 10.3389/fnut.2023.1174945 37469547 10.3389/fnut.2023.1174945 PMC10352804

[27] Yilmaz M, Atilla FD, Sahin F, Saydam G (2020) The effect of malnutrition on mortality in hospitalized patients with hematologic malignancy. Support Care Cancer 28(3):1441–1448. 10.1007/s00520-019-04952-5 31273503 10.1007/s00520-019-04952-5

[28] Szeja N, Grosicki S (2022) Nutritional status of patients with lymphoproliferative neoplasms before and after the first-line treatment. Expert Rev Hematol 15(1):83–91. 10.1080/17474086.2022.2035717 35099347 10.1080/17474086.2022.2035717

[29] Xiong J, Chen K, Huang W, Huang M, Cao F, Wang Y et al (2023) Prevalence and effect on survival of pre-treatment sarcopenia in patients with hematological malignancies: a meta-analysis. Front Oncol 13:1249353. 10.3389/fonc.2023.1249353 37869092 10.3389/fonc.2023.1249353 PMC10587577

[30] Staxen CS, Andersen SE, Pedersen LM, Poulsen CB, Andersen JR (2024) Nutrition and lifestyle-related factors as predictors of muscle atrophy in hematological cancer patients. Nutrients. 10.3390/nu16020283 10.3390/nu16020283 PMC10819894 38257176

[31] Surov A, Wienke A (2021) Sarcopenia predicts overall survival in patients with malignant hematological diseases: a meta-analysis. Clin Nutr 40(3):1155–1160. 10.1016/j.clnu.2020.07.023 32768316 10.1016/j.clnu.2020.07.023

[32] Anabtawi NM, Pasala MS, Grimshaw AA, Kharel P, Bal S, Godby K et al (2024) Low skeletal muscle mass and treatment outcomes among adults with haematologic malignancies: a systematic review and meta-analysis. J Cachexia Sarcopenia Muscle 15(3):1084–1093. 10.1002/jcsm.13446 38558541 10.1002/jcsm.13446 PMC11154774

[33] Capitão C, Coutinho D, Neves PM, Capelas ML, Pimenta NM, Santos T et al (2022) Protein intake and muscle mass maintenance in patients with cancer types with high prevalence of sarcopenia: a systematic review. Support Care Cancer 30(4):3007–3015. 10.1007/s00520-021-06633-8 34697674 10.1007/s00520-021-06633-8

[34] Wang M, Zhao A, Li M, Niu T (2023) Amino acids in hematologic malignancies: current status and future perspective. Front Nutr 10:1113228. 10.3389/fnut.2023.1113228 37032776 10.3389/fnut.2023.1113228 PMC10076797

[35] Bendellaa M, Lelièvre P, Coll JL, Sancey L, Deniaud A, Busser B (2024) Roles of zinc in cancers: from altered metabolism to therapeutic applications. Int J Cancer 154(1):7–20. 10.1002/ijc.34679 37610131 10.1002/ijc.34679

[36] Abraham A, Kattoor AJ, Saldeen T, Mehta JL (2019) Vitamin E and its anticancer effects. Crit Rev Food Sci Nutr 59(17):2831–2838. 10.1080/10408398.2018.1474169 29746786 10.1080/10408398.2018.1474169

[37] Cai M, Wu X, Jin H, Li Z, Li Y, Chen Z (2024) Association of serum folate concentrations with cancer mortality among patients with arthritis: a prospective cohort study. BMC Public Health. 10.1186/s12889-024-21004-8 39696139 10.1186/s12889-024-21004-8 PMC11657765

[38] Mądra-Gackowska K, Szewczyk-Golec K, Gackowski M, Woźniak A, Kędziora-Kornatowska K (2023) Evaluation of selected parameters of oxidative stress and adipokine levels in hospitalized older patients with diverse nutritional status. Antioxidants (Basel). 10.3390/antiox12030569 36978817 10.3390/antiox12030569 PMC10044703

[39] Rafiee P, Jafari Nasab S, Bahrami A, Rezaeimanesh N, Jalali S, Hekmatdoost A et al (2021) Dietary total antioxidant capacity and colorectal cancer and colorectal adenomatous polyps: a case-control study. Eur J Cancer Prev 30(1):40–45. 10.1097/CEJ.0000000000000577 32079892 10.1097/CEJ.0000000000000577

[40] Toorang F, Seyyedsalehi MS, Sasanfar B, Rashidian H, Hadji M, Gholipour M et al (2024) Dietary total antioxidant capacity and odds of lung cancer: a large case-control study. BMC Cancer 24(1):1196. 10.1186/s12885-024-12914-2 39333933 10.1186/s12885-024-12914-2 PMC11438408

[41] Ghanavati M, Clark CCT, Bahrami A, Teymoori F, Movahed M, Sohrab G, et al. (2021) Dietary intake of polyphenols and total antioxidant capacity and risk of prostate cancer: A case–control study in Iranian men. Eur J Cancer Care (Engl). 30(2). 10.1111/ecc.13364 10.1111/ecc.13364 33174661

[42] Odutola MK, van Leeuwen MT, Turner J, Bruinsma F, Seymour JF, Prince HM et al (2022) Associations between smoking and alcohol and follicular lymphoma ıncidence and survival: a family-based case-control study in Australia. Cancers (Basel). 10.3390/cancers14112710 35681690 10.3390/cancers14112710 PMC9179256

[43] Ahmadkhaniha R, Yousefian F, Rastkari N (2021) Impact of smoking on oxidant/antioxidant status and oxidative stress index levels in serum of the university students. J Environ Health Sci Eng 19(1):1043–1046. 10.1007/s40201-021-00669-y 34150292 10.1007/s40201-021-00669-y PMC8172765

[44] Ajebli M, Meretsky CR, Akdad M, Amssayef A, Hebi M (2024) The role of dietary vitamins and antioxidants in preventing colorectal cancer: a systematic review. Cureus 16(7):e64277. 10.7759/cureus.64277 39130946 10.7759/cureus.64277 PMC11315617

[45] Bahrami A, Shirani P, Sohouli M, Nasab SJ, Rafiee P, Naja F et al (2022) Dietary diversity score (DDS) and odds of colorectal cancer and adenoma: a case-control study. J Nutr Sci. 10.1017/jns.2022.30 35620763 10.1017/jns.2022.30 PMC9107998

[46] Cano-Ibáñez N, Barrios-Rodríguez R, Lozano-Lorca M, Vázquez-Alonso F, Arrabal-Martín M, Triviño-Juárez JM et al (2020) Dietary diversity and prostate cancer in a Spanish adult population: CAPLIFE study. Nutrients 12(6):1–14. 10.3390/nu12061694 10.3390/nu12061694 PMC7352258 32517184

[47] Ha K, Kim K, Sakaki JR, Chun OK (2020) Relative validity of dietary total antioxidant capacity for predicting all-cause mortality in comparison to diet quality indexes in US adults. Nutrients. 10.3390/nu12051210 32344879 10.3390/nu12051210 PMC7282024

